# Prevention of sclerosis around cannulated screw after treatment of femoral neck fractures with bioceramic nails: a finite element analysis

**DOI:** 10.1186/s12891-023-06677-3

**Published:** 2023-07-12

**Authors:** Yang Liu, Yugang Xing, Huifeng Shao, Xiaogang Wu, Yongsheng Ma, Wenming Yang, Qitai Lin, Pengcui Li, Yong He, Wangping Duan, Xiaochun Wei

**Affiliations:** 1 Department of Orthopaedics, Second Hospital of Shanxi Medical University, Shanxi Key Laboratory of Bone and Soft Tissue Injury Repair, No. 382, Wuyi Road, 030001 Taiyuan, China; 2 School of Mechanical Engineering, Hangzhou Dianzi University, Hangzhou, 310018 China; 3 Key Laboratory of 3D Printing Process and Equipment of Zhejiang Province, School of Mechanical Engineering, Zhejiang University, Hangzhou, 310027 China; 4 Institute of Biomedical Engineering, College of biomedical Engineering, Taiyuan University of Technology, Taiyuan, 030024 China

**Keywords:** Femoral neck fracture, Nails, Finite element analysis

## Abstract

**Purpose:**

Conventional cannulated screws (CS) are the main treatment method for femoral neck fractures (FNF). However, the rate of femoral head necrosis remains high after FNF treatment. The study aimed to compare the biomechanical features of different internal fixation materials for the treatment of Pauwel type III FNF to explore new strategies for clinical management.

**Methods:**

A new material was prepared by applying casting, freeze drying and sintering process. The independently developed calcium magnesium silicate ceramic powder and hydrogel solution were evenly mixed to obtain a high-viscosity bio-ink, and a bioceramic nail (BN) with high mechanical strength and high fracture toughness was successfully prepared. Four internal fixations were developed to establish the Pauwel type III FNF and healed fracture finite element models: A, three CSs; B, three BNs; C, two BNs and one CS; D, one BN and two CSs. Von Mises stress and displacement of the implants and femur were observed.

**Results:**

The measured Mg content in ceramic powder was 2.08 wt%. The spectral data confirmed that the ceramic powder has high crystallinity, which coincides with the wollastonite-2 M (PDF# 27–0088). The maximum von Mises stresses for the four models were concentrated in the lower part of the fracture surface, at 318.42 Mpa, 103.52 MPa, 121.16 MPa, and 144.06 MPa in models A, B, C, and D, respectively. Moreover, the maximum Von-mises stresses of the implants of the four models were concentrated near the fracture end at 243.65 MPa (A) and 58.02 MPa (B), 102.18 MPa (C), and 144.06 MPa (D). The maximum displacements of the four models were 5.36 mm (A), 3.41 mm (B), 3.60 mm (C), and 3.71 mm (D). The displacements of the three models with BNs were similar and smaller than that of the triple CS fracture model. In the fracture healing models with and without three CSs, the greatest stress concentration was scattered among the lowest screw tail, femoral calcar region, and lateral femur shaft. The displacement and stress distributions in both models are generally consistent. The stress distribution and displacement of the three healed femoral models with BNs were essentially identical to the healing models with three CSs. The maximum von Mises stresses were 65.94 MPa (B), 64.61 MPa (C), and 66.99 MPa (D) while the maximum displacements of the three healed femoral models were 2.49 mm (B), 2.56 mm (C), and 2.49 mm (D), respectively.

**Conclusions:**

Bioceramic nails offer greater advantages than conventional canulated screws after femoral neck fractures. However, the combination of bioceramic nails and CSs is more clinically realistic; replacing all internal fixations with bioceramic nails after the healing of femoral neck fractures can solve the problem of sclerosis formation around CSs and improve bone reconstruction by their bioactivity.

**Supplementary Information:**

The online version contains supplementary material available at 10.1186/s12891-023-06677-3.

## Introduction

Approximately 1.5 million hip fractures occur worldwide each year, and this number is predicted to increase to 6.3 million by 2050 [1]. Additionally, the mortality rate is high [2, 3]. As one of the most common types of injuries in orthopaedics, femoral neck fractures (FNF) account for 14% of all hip-related fractures and over 50% of proximal femoral fractures in the United States [4]. Solid internal fixation and anatomical reduction are significant factors for successful fracture healing. A single fixed-angle screw with a lateral large plate or cannulated screws (CSs) (i.e., dynamic hip screw, DHS, AKA a sliding hip screw) is currently the surgical approach for treating FNFs. Laboratory and biomechanical studies have shown that, while sliding hip screws have greater resistance to shearing forces, especially in displaced, and unstable fracture types, which are the main reason for implant failure, multiple CSs improve resistance to rotational forces [5–7] (the second leading cause of implant failure) and are less invasive [8, 9]. Additionally, tension screws are required to apply pressure to the fracture end before locking screws are used. Therefore, there are obvious limitations to clinical applications [10]. It should be noted that the risk factors of femoral neck fractures treatment failure depend on multitude of factors, including the implant type, time from the trauma to surgery, bone quality, the grade of dislocation, etc. Further, the final result does not completely depend on the treatment method.

The need for reoperation after these interventions is still high (10.0–48.8%) and has not essentially changed over the past 30 years [11]. A high reoperation rate has raised controversy regarding the best treatment for FNF [12]. Therefore, we continue to explore the next generation of effective internal fixations. The advantages and disadvantages of each implant from laboratory and biomechanical studies have inspired the design of a new hip fracture implant and a new surgical procedure.

**Fig. 1:**
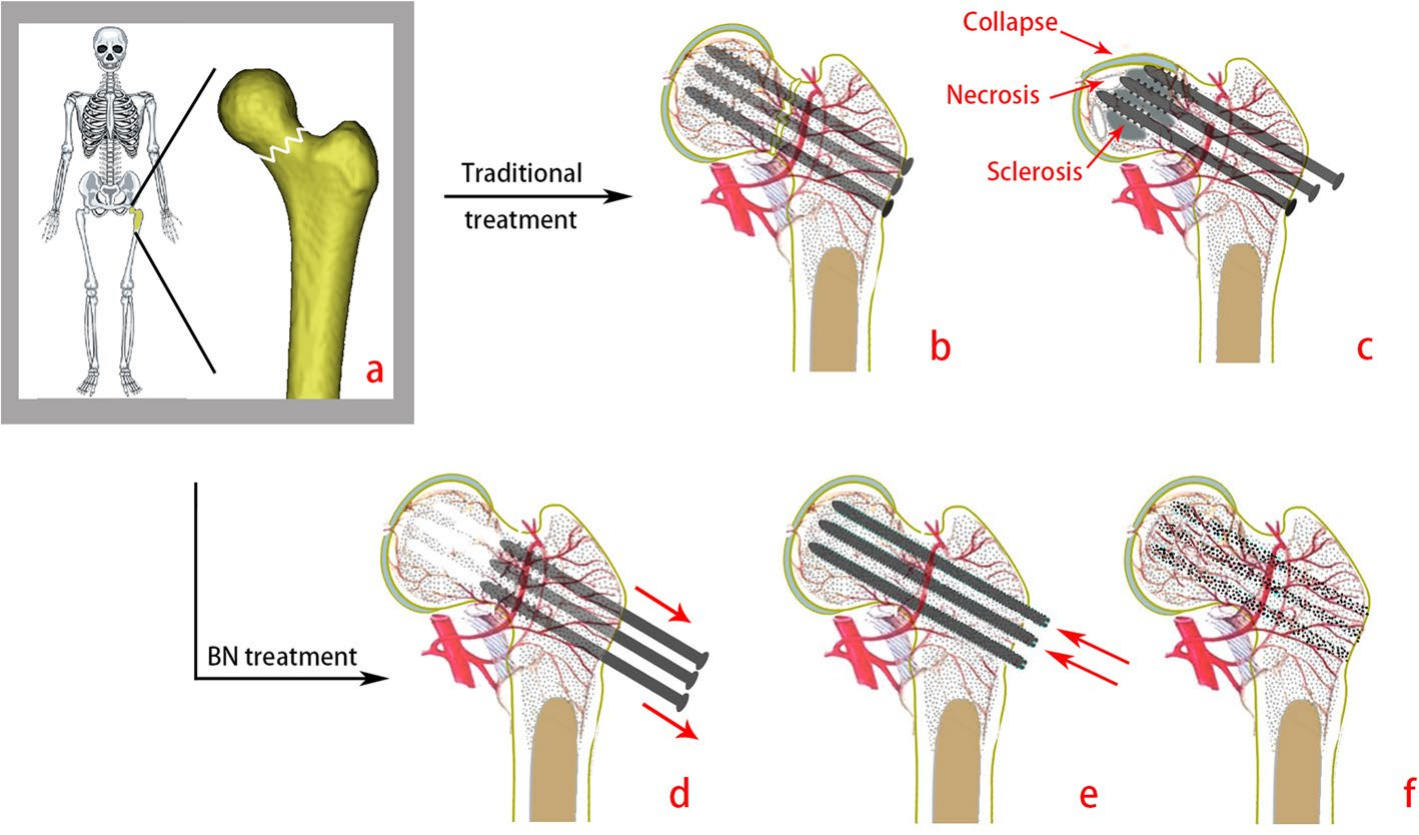
Two treatment methods for FNF. **a** the whole skeleton (left), the bone with femoral neck fracture (right), **b **treatment with CSs, **c **Sclerosis, necrosis and collapse found in the femoral head./ the result of traditional treatment, **d **removal of CSs before sclerosis occur, **e **put the bioceramic nails in, **f **the result of traditional treatment

Previously our team has determined that regardless of whether implants are removed, there is risk of collapse of the femoral head, for as long as sclerotic bone around the screw paths persist following healing of FNFs. In addition, as we concluded in a clinical work, years after removal of the internal fixation material, no effective tissue filling develops in the screw paths [13]. Preventing the formation of osteosclerotic areas is critical. If a new calcium-magnesium-silicate ceramic nail is implanted into the screw path after the healing of FNF and before the formation of osteosclerotic area around screw paths, it can prevent fracture complications by accelerating the osteogenic vascularization and restoring the mechanical properties of the femur in advance (Fig. 1). Although several studies have suggested that these two types of materials have certain advantages, they have not been consistently recognized, and there has been no effective finite element experiment to assess the mechanical stability of the two materials. Therefore, we modelled the FNF and compared the mechanical differences between bioceramic nails (BNs) and CSs using a finite element analysis (FEA).

Despite this, in clinical practice, structures such as BN threads cannot be made directly with three-dimensional (3D) printing as printing threads are not mechanically strong enough [14]; hence, it is impossible to solely screw BNs into the femur, but the screw paths can only be filled with bioceramic material after the CSs have been removed. Therefore, there are obvious limitations to the clinical applications of this method. If the mechanical properties of the finite element results of the BNs researched in the previous section are not good, it is possible to use the BNs in combination with the CSs by replacing only one or two CSs after fracture healing, resulting in a reduced formation of the sclerotic bone, which will also decrease the stress concentration of the femoral head. Therefore, the stress and displacement of single BN with two CSs and double BNs with one Cs models were also observed by FEA.

**Fig. 2:**
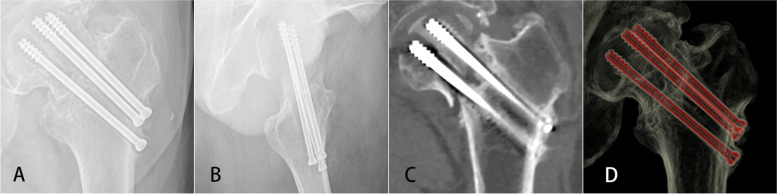
A case of unhealed femoral neck fracture. **A** Coronal X-ray, **B** sagittal X-ray, **C** coronal CT, **D** sagittal CT

Regarding the issue of sclerosis, we have considered whether the bone trabecular around screws is already sclerosed before it is healed. If so, together with the impossibility of treating the FNFs directly with the BN, even if the fracture heals and is subsequently replaced with a BN, sclerosis cannot be prevented. However, in clinical practice, we found some cases (Fig. 2) where fractures have not fractured after years of internal fixation treatment, no high-density formation around screws were observed on CT, and the trabecular bone had not started reconstruction. In fact, sclerosis of the trabeculae must be analysed together with the process of fracture healing [15]. The healing of fractures and bone defects normally follows an orderly series of events, including the formation of a hematoma, an initial stage of inflammation, development of soft callus, formation of hard callus, and finally bone remodeling. Therefore, it is only after the fracture of the femoral neck has healed that the bone trabeculae are reconstructed and the sclerotic bone is formed. If the patient waits until the fracture has healed and subsequently immediately replaces the CS with a BN, a certain mechanical and biological advantage can be guaranteed. A FNF healing model was constructed to verify the effect of removing the CSs on stress. If the effect was small, the CSs were replaced one by one until they were all replaced by BNs, and the mechanical differences between the three models are compared using FEA.

FNFs are often vertical and unstable, with a higher shear angle and greater shear force. Even without weight-bearing, the contraction force of the abundant hip muscles is sufficient to generate a considerable shear force between the ends of the fracture, resulting in instability between ends. The shearing force is conducive to the healing of the fracture, which is inclined to hip varus displacement and postoperative complications, such as nonunion, fixation failure, and avascular necrosis, which all have a high incidence [16]. Current treatment modalities have not been unified for severe unstable FNFs. FNF can be classified according to different standard, namely the AO group, Garden, and Pauwell [17]. As the first biomechanical classification of FNF, the Pauwell classification has been widely used in the literature and preoperative guidelines. This classifies FNFs into three types based on fracture direction: the angle between the distal fracture line and the horizontal line [18]. When the fracture pattern is more vertical (Pauwell III, namely > 50°), the femoral neck is subjected to greater shear than compression forces, which is associated with a higher rate of varus instability and fracture healing complications [19, 20]. Pauwel type III FNF is a better test for mechanical properties of internal fixation materials. Thus, in the present study, a Pauwell III type fracture was applied as the fracture model.

In the past decade, the design of medical device and biomechanical assessment have benefited from computer modelling studies, especially FEA [21]. FEA can provide quantitative biomechanical information about orthopaedic implants and improve our understanding of the mechanical behaviours of implants and interactions of bone implants [22]. Finite element models can be used to access the biomechanical properties of internal fixation to ensure the distribution of stress in bone and implants can be predicted.

In summary, this paper aimed to analyze the mechanical performance of BNs and traditional CSs in the treatment of unstable FNFs, demonstrate the biomechanical stability of combined implants, and to offer a theoretical foundation for the clinical application of new-type BNs.

## Materials and methods

### Production of bioceramic nails

**Fig. 3:**
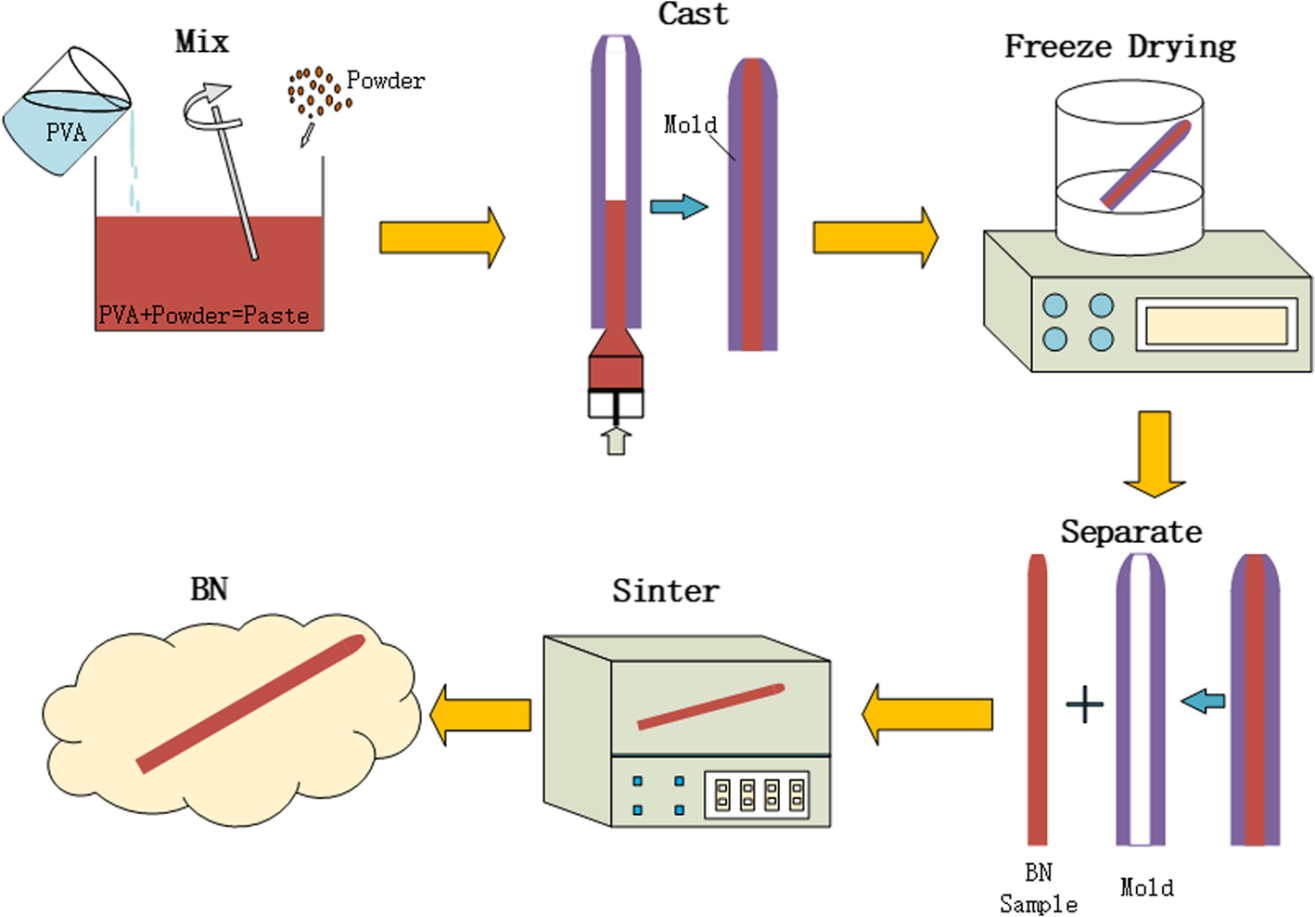
Schematic illustration of the manufacturing process of BNs. (1) Mixing ceramic powders with PVA solution, (2) Pouring the paste into the mold, (3) Freeze drying process in the lyophilizer, (4) Separating the BN sample from the mold, (5) Sintering in the furnace

All BNs were prepared using casting, freeze drying and sintering process. The independently developed calcium magnesium silicate ceramic powder and hydrogel solution were evenly mixed to obtain a high-viscosity bio-ink, and a bioceramic material with high mechanical strength and high fracture toughness was successfully prepared (Fig. 3). The bioceramic nails were made into cylinders 10 mm long and 7.3 mm in diameter. Subsequently, the modulus of elasticity of each sample was measured using the universal testing machine with a 0.5 mm/min crosshead speed. (model 1144, Instron; Norwood, MA).

The effects of various sintering temperatures, scaffold porosity, and other parameters on the mechanical properties of artificial bone scaffolds have been considered [23]. Using this scaffold material, a BN in line with the internal fixation screw paths of the FNF was designed and manufactured. The scaffold materials possess good biological activity, demonstrated by the calcified layer on the surface of bioceramic material in simulated body fluid, controllable degradation by degradation experiments in Tris-Hcl solution, cytocompatibility that promotes cell adhesion, proliferation, and differentiation by cell experiments [24], and good osteoinductive and osteogenic properties by defect repair experiments in vivo. Improving the spatial structure of bioceramic scaffolds can significantly improve the osteogenic vascularization of scaffold materials in vivo [25]. This material is also commonly used in spinal diseases and has high frictional properties to prevent implants from being pulled out [26]. The phase composition of the ceramic powders was verified by X-ray diffractometer (XRD; Rigaku Co., Japan) at 40 kV/40 mA. Data were collected between 10^o^ and 60^o^ with a step of 0.02^o^/2θ and a dwell time of 1.5 s to identify any crystalline phase of the powders. The inorganic ion content in ceramic powder was measured by inductively coupled plasma-optical emission spectrometry (ICP-OES; Thermo Icap 6000 series). The powders were observed by using the scanning electron microscopy (SEM, S-4800; Japan) at 10 kV. The particle size distribution was analyzed by dynamic light scattering (DLS, Malven Instrument 2000) in purified water medium.

### FEA of the Pauwell III-type FNF

A 31-year-old healthy male volunteer without history of hip joint or systemic disease was recruited. The femur was scanned with a layer thickness of 0.5 mm using the GE Revolution CT scanner (GE Healthcare, Chicago, IL). The scanning protocols were 80 kV and 450 mA. Thereafter, CT images were stored in DICOM format files into the medical 3D reconstruction Mimics 21.0 software (Materialise, Leuven, Belgium). According to the gray value of bone tissue and regional segmentation, the 3D models of cancellous bone and cortical bone were reconstructed by Boolean operation. After that, models were exported as a file in STL format.

**Fig. 4:**
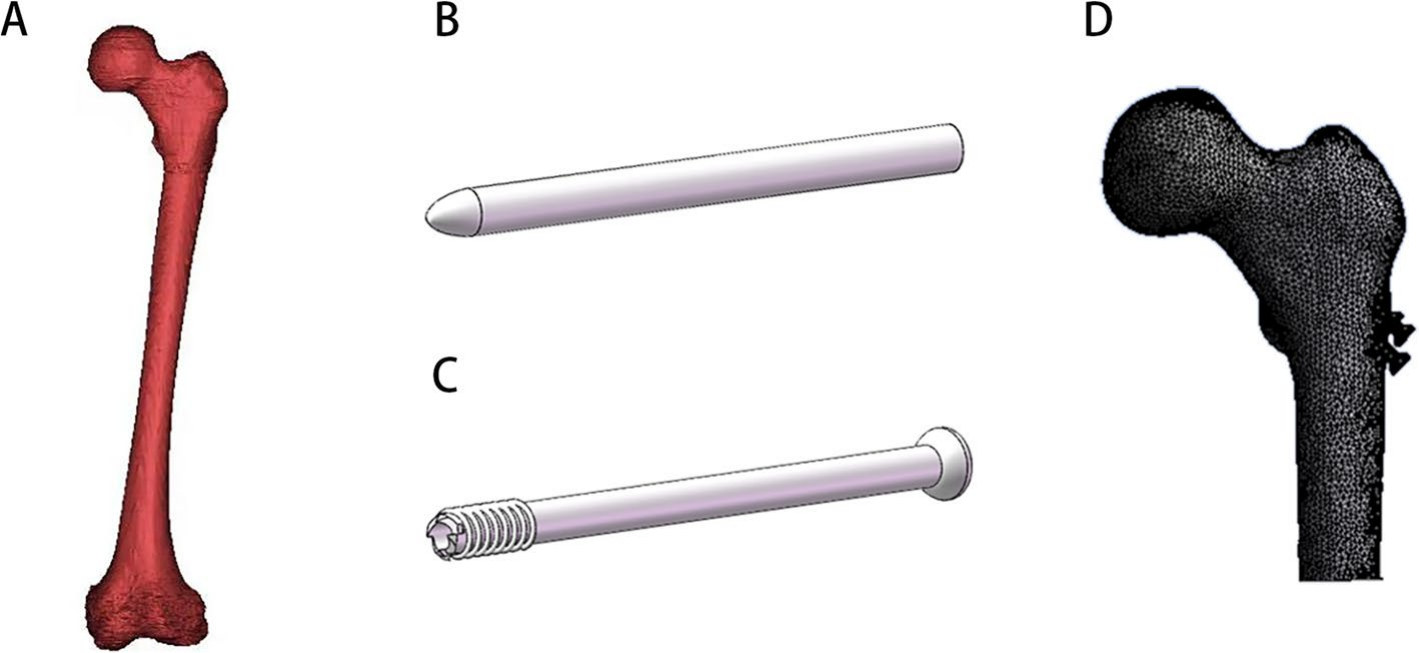
Finite element modelling process. **A** Rebuilt 3D model of femur from CT of patient, **B**, **C** Bioceramic nail model and CS model produced in SolidWorks, **D** Finite element mesh model of proximal femur

The model was imported into Geomagic Studio 2017 software (3D System Inc., Rock Hill, SC, USA) for smoothing, meshing, and fitting surface, and then exported as an STEP file. This study employed a Pauwell III type FNF (≥ 50°). We created a cutting plate across the center of the femoral neck at an angle of 40° to the sagittal plane of the pivot axis. The femoral neck was divided using the cutting plate to simulate the Pauwell III FNF (Fig. 4). Based on the engineering geometry data and clinical fixation programming, the models of CSs (7.3 mm in diameter, 16 mm in thread length, 85 mm in total length) and BNs (7.3 mm in diameter and 85 mm in total length) were generated using Solidworks software (Solidwords Corp., Waltham, MA) [27].

**Fig. 5:**
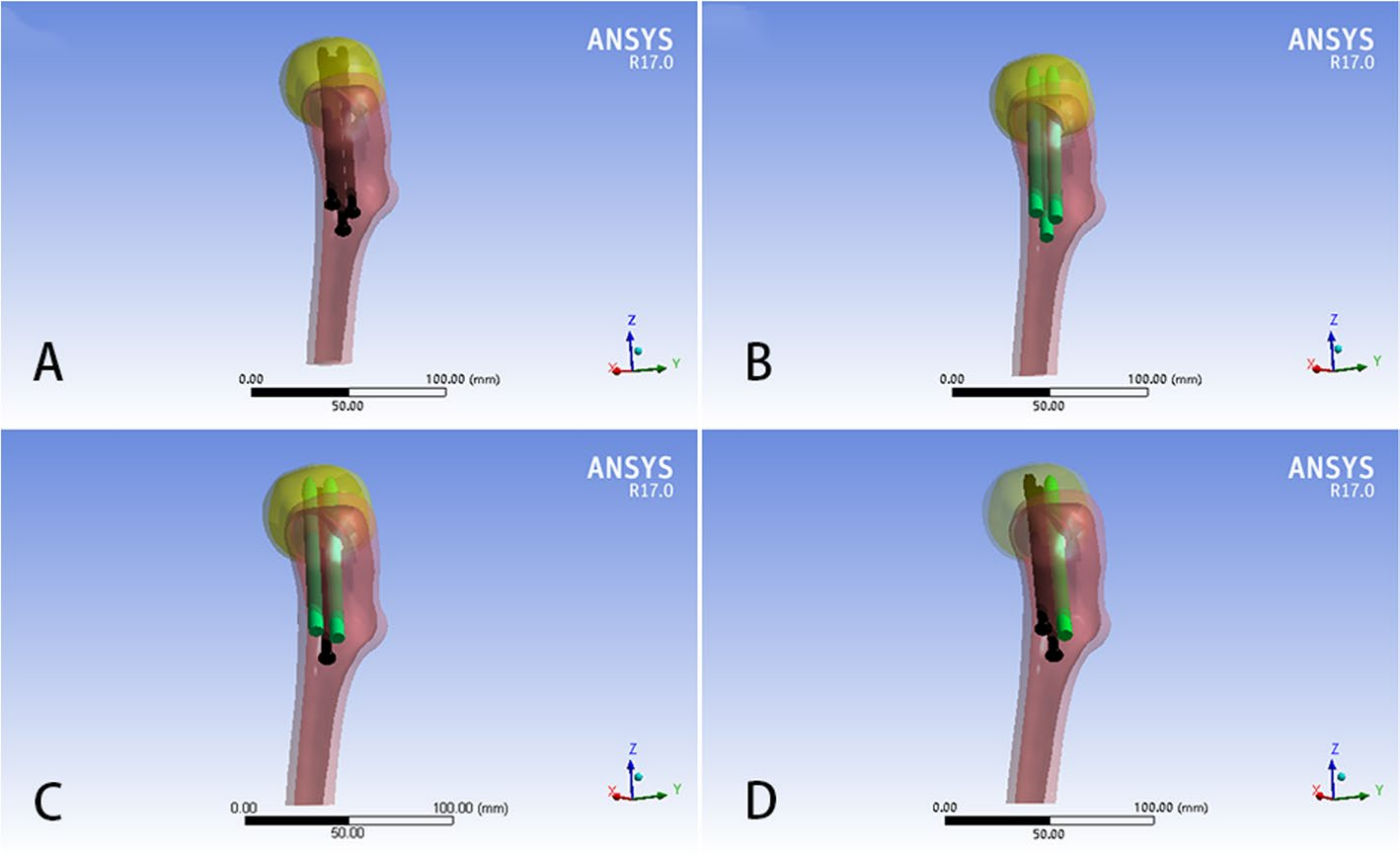
Four internal fixation models. Model **A**: three CSs, model **B**: three BNs, model **C**: two BNs and one CS, model **D**: one BN and two CSs

Following the surgery reported in the literature, the three parallel implants were arranged in an inverted triangle. Four types of internal fixation (A, three CSs; B, three BNs; C, two BNs and one CS; D, one BN and two CSs, Fig. 5) were constructed at an angle of 135° to the longitudinal axis of the femur [28]. Osteotomy was performed at the mid-femoral stem to create a model of the proximal femur (Fig. 4). After meshing all models using HyperMesh 11.0 (Altair Engineering, Inc. USA), a finite element analysis was performed using the ANSYS17.0 (Ansys Inc, Canonsburg, PA, USA). The modulus of elasticity and Poisson’s ratio of various materials are listed in Table 1 [29, 30].

**Table 1:** Material properties of the various components in the models

Item	Elastic modulus (MPa)	Poisson’s ratio
Femoral Cortical bone	16,800	0.30
Femoral Cancellous bone	840	0.20
Cannulated screw Bioceramic nail	20,600 9000	0.30 0.25

Regarding the boundary conditions and loading force settings, during the analysis, all nodes on the distal femoral surface were restricted to 0 degrees of freedom to prevent rigid body motion [31]. Mesh convergence studies showed that when the number of femur element was increased to 276,579, the incremental displacement of the model as the mesh changes is less than 4%, meaning that the change in displacement is almost negligible. The fracture healing models were discretized into 276,401 elements with 434,497 nodes. Frictional contact was used to describe the interaction between the fracture ends as well as between bone and implants. The friction coefficient between the CS and femur was 0.2 [32]. Fracture ends were defined as completely broken and are considered to have been repositioned. The coefficient of friction was 0.46 [33]. According to Van Houcke, joint forces during single-leg stance were loaded on the corresponding cartilage surface of the femoral head with a one-cycle force value of 1800 N [33]. Ten tetrahedral nodal units were automatically generated. The various materials in the model were assumed to be homogeneous and isotropic linear elastic materials [34, 35]. Finally, the von Mises stress and displacement of the implants and femur were observed.

## Results

### The results of ceramic powders and BN

**Fig. 6:**
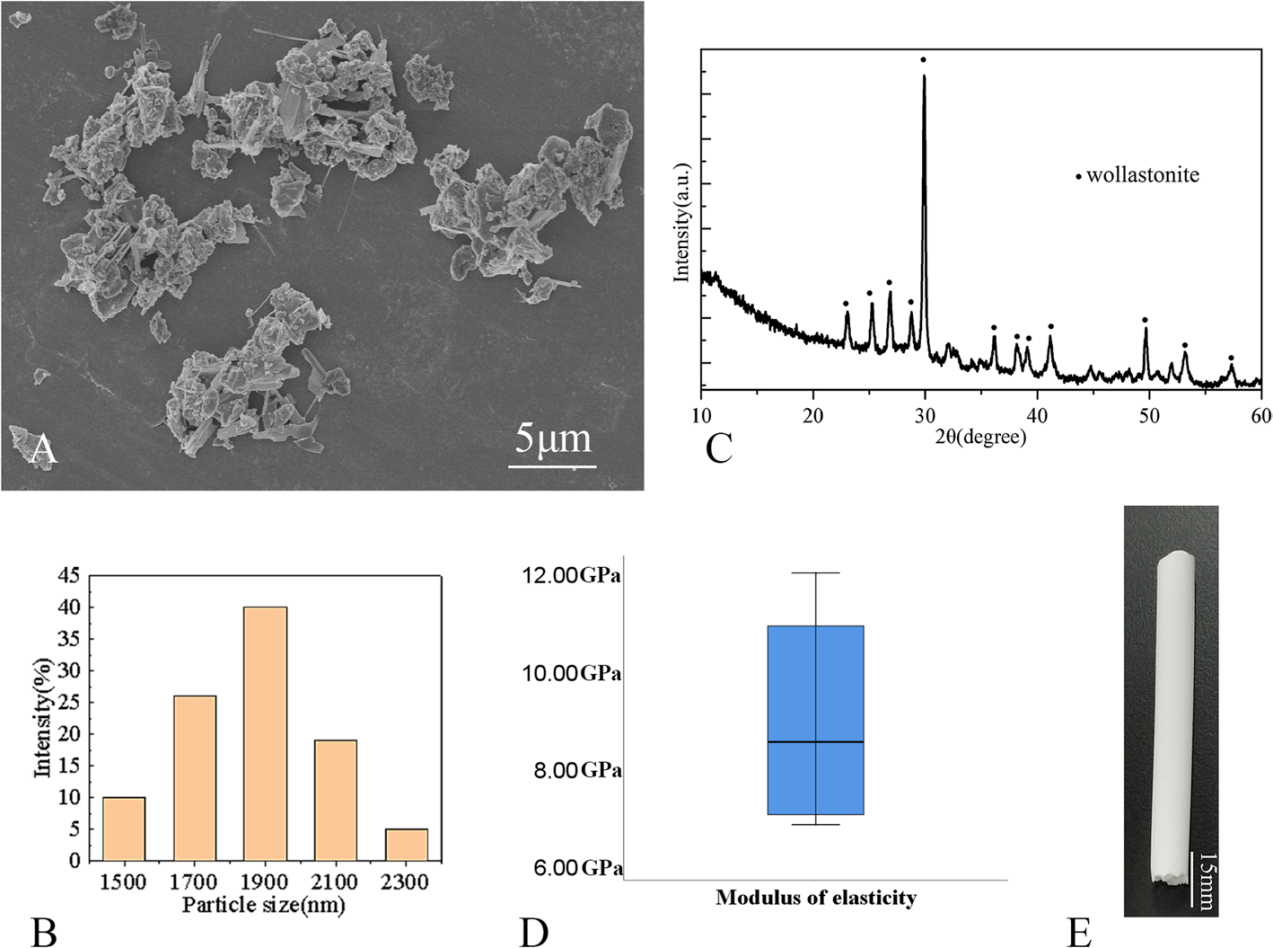
The results of ceramic powders and BN. **A** the SEM observation results, (**B**) Evaluation of the particle size distribution conducted using DLS, (**C**) the XRD pattern of the ceramic powders, (**D**) Box plot of modulus of elasticity measurement results for bioceramic nail, (**E**) appearance of the bioceramic nail

Figure 6A shows the SEM observation results. It can be seen that the particle size of ceramic powders after calcination are all less than 5 μm. Evaluation of the particle size distribution conducted using DLS revealed a narrow size distribution in the range of 1500 − 2300 nm for ceramic powders (Fig. 6B). Also, it is worth noting that the measured Mg content in ceramic powder was 2.08 wt%, which was close to the theo-retical data (2.12 wt%) calculated by the 10% Ca substitution by Mg in the stoichio-metric wollastonite. The XRD pattern of the ceramic powders are presented in Fig. 6C. The spectral data confirmed that the ceramic powder has high crystallinity, which coincides with the wollastonite-2 M (PDF# 27–0088). The final modulus of elasticity of the bioceramic nails was found to be 8.95 ± 2.41 GPa (Fig. 6D). A finite element model was constructed using 9 GPa as the value for the bioceramic nail (Supplementary Fig. 1). Figure 6E showed good straightness and uniformity of BN.

### The results of fracture models

**Fig. 7:**
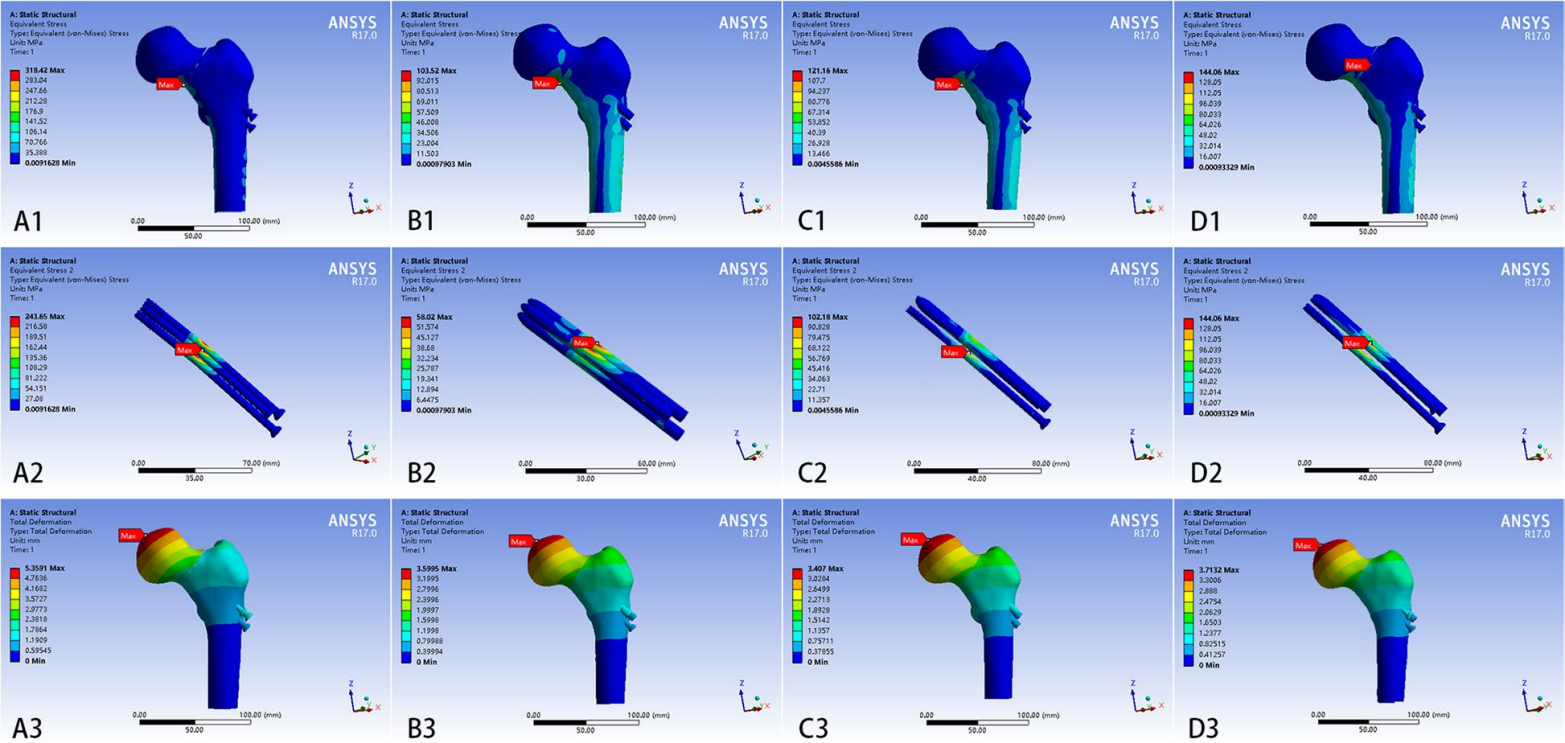
The finite element result of femoral neck fracture models with four types of internal fixations. (A) stress distribution and displacement of the femoral neck fracture model with three CSs and the implants of the models, (B) stress distribution and displacement of the femoral neck fracture healing model with three bioceramic nails and the implants of the models, (C) stress distribution and displacement of the femoral neck fracture healing model with one CS and two bioceramic nails and the implants of the models, (D) stress distribution and displacement of the femoral neck fracture healing model with two CSs and one bioceramic nails and the implants of the models

The maximum von Mises stresses in the four models were concentrated in the lower part of the fracture surface at 318.42 MPa, 103.52 MPa, 121.16 MPa, and 144.06 MPa, respectively (Fig. 7). The stresses in the four models were mainly distributed in the lower part of the femoral neck and the medial and lateral parts of the femoral shaft. Moreover, the stresses in the modes with B, C, and D internal fixation types were dispersed and homogeneous compared to model with A type internal fixation type. The maximum von Mises stresses of implants for four models were concentrated near the fracture end at 243.65 MPa and 58.02 MPa, 102.18 MPa, and 144.06 MPa, respectively (Fig. 7). The stress for implants were much greater in model with A internal fixation type than in the other three models. Moreover, the maximum stress in models with A and D internal fixation types were located anteriorly above the inverted triangle, the maximum stress in model with B internal fixation type was located posteriorly above the inverted triangle, and the maximum stress in model with C internal fixation type was on the CS. The most significant displacement of all the models occurred at the femoral head. The maximum displacements of the four models were 5.36 mm, 3.41 mm, 3.60 mm, and 3.72 mm, respectively (Fig. 7), while the displacements of the fracture ends of the four models were approximately 2.98 mm, 1.89 mm, 2.00 mm, and 2.06 mm, respectively. The models with BCD internal fixation types had similar displacements at each site, and all displacements were significantly smaller than that of model with A internal fixation type.

### Healing models with and without three CSs

**Fig. 8:**
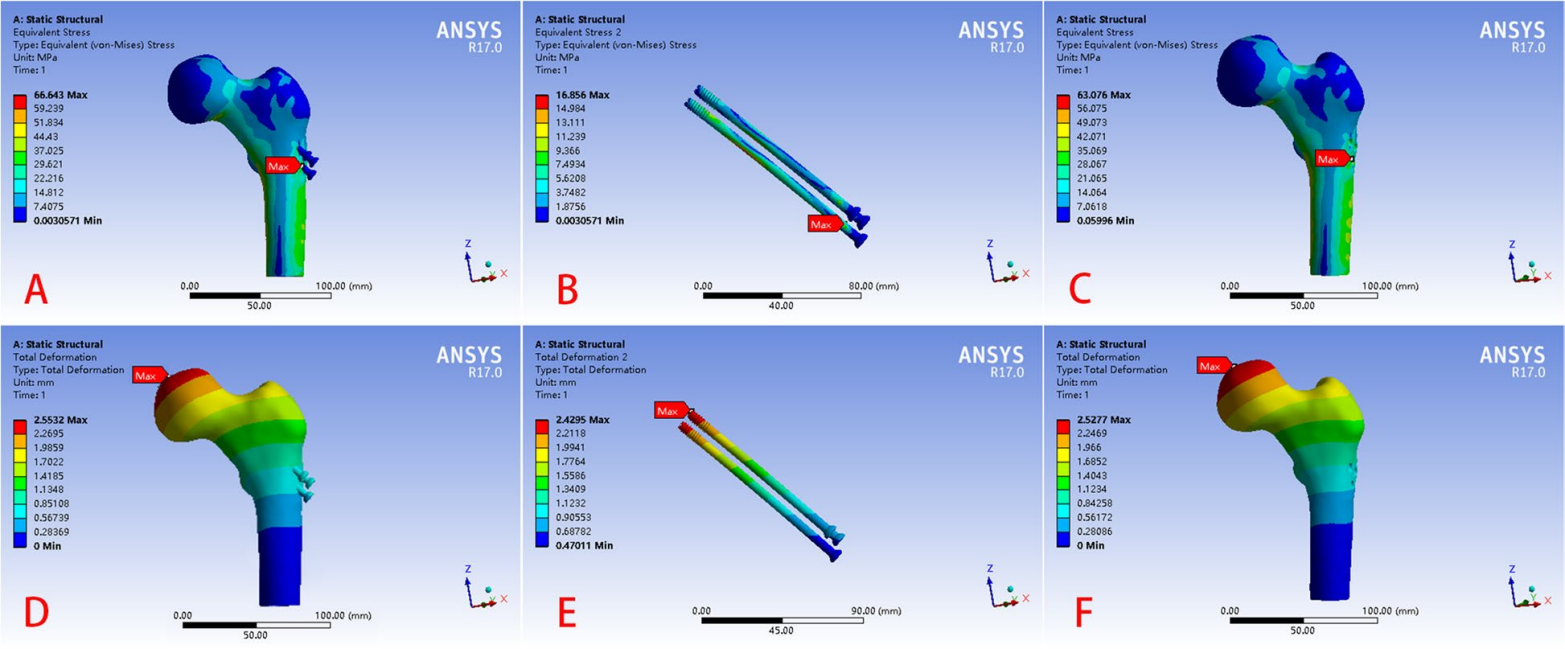
Finite element results of the healing model before and after removal of the implants (**A**) stress distribution of the femoral neck fracture healing model with three CSs, **B** the stress distribution of the implants in A, **C** stress distribution of the the healing model, **D** the displacement of the femoral neck fracture healing model with three CSs, **E** displacement of the implants in D; **F** displacement of the the healing model

In the fracture healing models with and without three CSs, the greatest stress concentration was scattered among the lowest screw tail, femoral calcar region, and lateral femur shaft. The maximum von Mises stresses in the two models were 66.64 MPa and 63.08 MPa, respectively (Fig. 8); the maximum displacements in the two models were 2.55 mm and 2.53 mm, respectively. The displacement and stress distributions in both models were generally consistent. The implants had a maximum von Mises stress of 16.86 MPa (Fig. 8B) and displacement of 2.43 mm.

### Models with three types of mixed implants

**Fig. 9:**
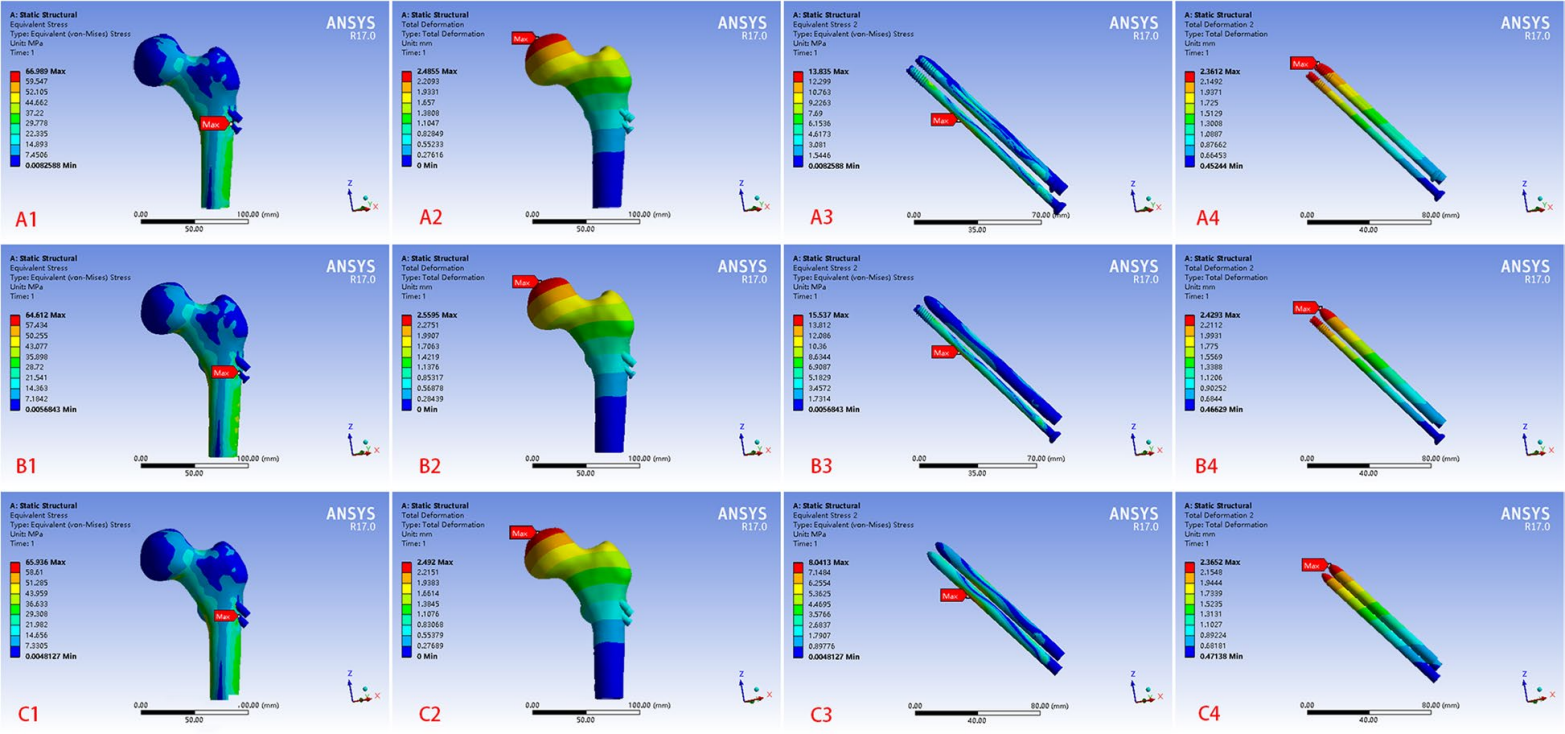
The finite element result of femoral neck fracture healing models with four types of internal fixations. A stress distribution and displacement of the femoral neck fracture healing model with two CSs and one bioceramic nail and the implants of the models, (B) stress distribution and displacement of the femoral neck fracture healing model with one CS and two bioceramic nails and the implants of the models, (C) stress distribution and displacement of the femoral neck fracture healing model with three bioceramic nails and the implants of the models

The von Mises stress distribution and displacement of the three healed FNF models with BCD internal fixation types were essentially identical to the three healing models with three CSs. The maximum von Mises stresses were 66.99 MPa, 64.61 MPa, and 65.94 MPa; and the maximum displacements were 2.49 mm, 2.56 mm, and 2.49 mm, respectively (Fig. 9). The stress of internal fixation for the three healing models were different compared to those of the fracture models. The maximum stress on the internal fixation in all healed models was all located in the lower implant.

## Discussion

The bone is a living organ that is reconstructed and shaped for its load capacity to meet the need of an external mechanical environment [36]. The spatial distribution of bone trabeculae within the femoral head is closely related to mechanical adaptation, which originated from the observation that the orientation of principal stresses matches the alignment of the cancellous bone microarchitecture [37]. The long-term high stresses around the implants lead to the reactive growth of bone trabecula in this region. However, when an increase in bone mass does not compensate for long-term high stress stimulus, bone reconstruction is hindered. This is why the growth in the screw paths stops for many years after internal fixation treatment. Vesterrnark et al. [38] found a characteristic bony structure, the sclerotic bone rim (SB rim), which isolates the implants from the marrow cavity in revision surgery for failed hip replacement. After removing the internal fixation, the osteogenic substance cannot pass through this structure, further preventing bone growth in the screw paths. The sclerotic area is a partial biological response to the foreign body of the implants. In this paper, we used a 3D printer and sintering process to create 3D porous BNs with manageable internal pores and excellent HA layer deposition sedimentary capabilities. The degradation properties of the BNs were matched with the growth rate of the regenerated bone tissue. The modified bilayer printing technique increases the size of the lateral pores and accelerates new bone grow.

If the changes observed in bone density in the long-term after FNF are interpreted in terms of biomechanical properties, it is clear that these changes are due to a different load transfer in the healthy femur compared to the implanted model. The goal of internal fixation treatment is to achieve a physiological transfer from the femoral head to the metaphyseal cortex as in the original load femur to avoid stress shielding. Clinical treatment advocates the use of CSs with high stiffness and modulus of elasticity for fixation of the fracture, providing a stable mechanical environment for the bone in the short-term and preventing reinjury. However, because the stiffness of the CS is greater than the stiffness of the bone tissue and the stress distribution in the proximal femur is greatly reduced during stance, there is a loss of bone mass. In the later stages of fracture recovery, bone tissue can become osteoporotic due to insufficient mechanical stimulation and is often prone to re-fracture [39–41]. The longer the fixation, the worse the mechanical properties of the bone.

While the advantages of screw path implants in mechanics have been mentioned above, a study demonstrated that the presentation of a sclerotic region around the screw path increases the risk of femoral head collapse due to stress concentration and prevents sclerosis formation from becoming critical [13]. We believe that the clinical outcomes of the patients can be improved by selecting appropriate implant materials and optimizing surgical procedures. Thus, the mechanical properties of nails made of this new material were also investigated in this study. From the section of fracture model with B internal fixation, implants assumed a relatively small amount of stress, that is, about half the stress of the model with three CSs. Compared to the sliding hip screw (SHS) internal fixation system, prior studies showed that the use of bioceramic nails has resulted in a more uniform stress distribution in the overall femoral model, according to previous studies [42]. We assumed that this is because the modulus of elasticity of BN is only 9 GPa while the coefficient of friction is relatively high. The stress distributed in cancellous bone is relatively uniform, which can reduce the stress shielding and enable osteogenic reconstruction in response to stress. Moreover, bioceramic composite nails gradually degrade and are replaced by bone tissue over time. The mechanical support of nails gradually diminishes, reducing the biological response of the cancellous bone to the CS foreign body. These factors prevent osteoporosis and the formation of sclerosis. Ultimately, it is possible to reduce the occurrence of osteonecrosis and collapse of the femoral head. Additionally, from the sections of fracture models with A and B internal fixations for implants, it seems that the maximum bearing position is not always on the same implant, and the position has to be considered depending on implant characteristics.

After FNF, the ideal relationship of the fracture ends is that they should achieve anatomical reduction (i.e., good alignment of the fracture and restoration of the original anatomical position). In fact, the main reason why many surgeons do not remove internal fixation is that they do not want to disrupt the blood supply to the femoral head, which increases the possibility of necrosis and collapse. It is clear from section of fracture models that traditional CS fixation has an unstable alignment of the fracture end and has a large displacement, which tends to disrupt the blood supply to the femoral head. Regardless of whether the BNs are used alone or in combination with CSs, the entire displacement is considerably less than that of fracture model with A internal fixation. Therefore, it is more advantageous to use BN in clinic setting.

In the first operative plan, where BNs are used during surgical procedures for the treatment of fractures, a minimum of one CS must be left in place intraoperatively because of the immaturity of 3D printing of BNs, which cannot be used directly under pressure. This still carries the risk of peri-implant sclerosis, but compared to three CSs, this protocol significantly reduces the volume of sclerotic bone formed and its hazards. As CSs are gradually replaced with BNs, less stresses are placed on implants, and stresses become more uniform throughout the proximal femur. Moreover, the maximum stresses on the entire femoral model were reduced by more than half as long as the BN is present. In the second operative plan of replacing the BNs after fracture healing, stress distribution in the models was similar for all four combinations of internal fixation and the stresses in the internal fixation were low, again demonstrating that internal fixation can be removed after healing. In contrast to the results in the section of fracture models, the maximum stresses in the internal fixations in healed models were all located in the lower implant. After verifying that the mechanical effects of the four combinations of internal fixation are essentially the same, we determined that replacing all CSs with BNs after the healing of the FNF is the best approch to prevent sclerosis around the screw after FNF. This approach not only ensures the ability of internal fixation to take up stress after femoral neck fracture without increasing the risk of endophytic failure, but also reduces the formation of sclerosis around CSs, and subsequently reduces the occurrence of femoral head necrosis.

Various biomaterials have been used as clinical bone implants made from numerous materials, including carbon fiber, titanium, and magnesium [43]. Several studies have reported that although the biomaterials used to produce implants provide adequate mechanical support, there is no direct osseointegration between these materials and the host bone, and fibrous tissue forms between the host bone and the material [44]. Biodegraded magnesium alloys have been clinically used in hallux vaglus, wrist fracture, and FNF, and have shown good mechanical properties and biosafety [45]. Mg is a promising application for skeletal tissue engineering because of its important role in osteoblast adhesion [46], differentiation, proliferation, and bone mineralisation. It can also stimulate new bone formation [47]. Therefore, we proposed the ionic modification of silicates to balance their mechanical and biomedical properties.

Biodegradable bioactive materials are a prospective direction for orthopaedic internal fixation and have a very extensive range of applications. According to CT images, 3D models is reconstructed, and the clinical need for bone defect repair data is used as a guide to provide repair materials. This personalized repair material has excellent mechanical properties, in vitro bioactivity, and in vivo osteogenesis, providing excellent mechanical properties and good bioactivity for cancellous bone. In the practical condition of FNF repair, despite the inability of BNs to completely replace CSs, the combined application of CSs and BNs prevents bone sclerosis and retains sufficient mechanical support in the early stage of fracture healing. Additionally, it accelerates the bone growth of screw paths in the later stage of fracture healing, ultimately preventing femoral head necrosis or collapse. This study demonstrates the great potential of 3D printing in the personalized repair of bone defects, and lays the foundation for the subsequent manufacture of clinical artificial bone that meets individual needs of shape and performance. Owing to the limitations of 3D printing, the threads of the BN could not be built to imitate the screw-in surgical procedure of CS, which could be improved by transforming the top structure of BN in the future. However, it is unclear whether these changes in implant and design in the biological body can help avoid stress shielding and achieve better transfer of load to the proximal femur. In addition, there are multiple ways by which CS can be configured for FNF, such as inverted triangle and posterior triangle, and the physiological load is under the gait cycle, which also requires additional and more refined biomechanical analysis.

## Conclusion

In conclusion, after femoral neck fractures, models containing BNs offer greater advantages than conventional CSs in that the stresses are distributed and more uniform and that the displacement of the fracture end is decreased. However, the combination of BNs with CSs is more clinically realistic as BNs cannot be used directly under pressure. This combination also limits the incidence of sclerosis to a certain extent; replacing all internal fixation with a BN after healing of the femoral neck fracture would solve the problem of sclerosis around the CS and improve bone reconstruction through its bioactivity.

## Supplementary Information

**Additional file 1:** **Supplementary Figure 1.** Results of modulus of elasticity measurements onbioceramic nails.

## Data Availability

The datasets used and/or analyzed during the current study available from the corresponding author on reasonable request.
