# Supplementary material for: Prevention of sclerosis around cannulated screw after treatment of femoral neck fractures with bioceramic nails: a finite element analysis
Source: BMC Musculoskelet Disord. 2023 Jul 12;24:569. doi: 10.1186/s12891-023-06677-3 (PMC10337165; doi:10.1186/s12891-023-06677-3)
Supplement: Supplementary file 1 — Additional file 1: Supplementary Figure 1. Results of modulus of elasticity measurements onbioceramic nails. [file 12891_2023_6677_MOESM1_ESM.docx]

Supplementary Figure 1. Results of modulus of elasticity measurements on bioceramic nails


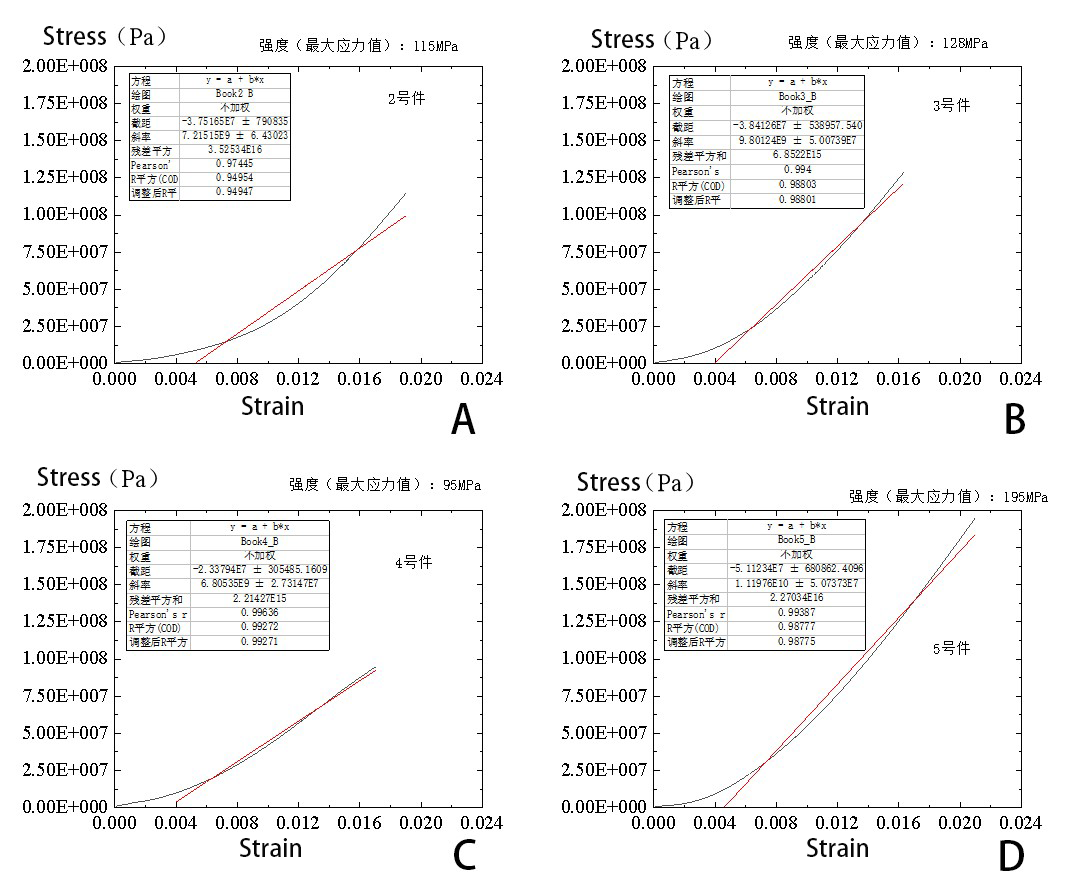


Stress-strain curves for each bio-ceramic nail specimen when tested with the universal testing machine.
